# Collaborator of alternative reading frame protein (CARF) regulates early processing of pre-ribosomal RNA by retaining XRN2 (5′-3′ exoribonuclease) in the nucleoplasm

**DOI:** 10.1093/nar/gkv1069

**Published:** 2015-11-03

**Authors:** Shigeko Sato, Hideaki Ishikawa, Harunori Yoshikawa, Keiichi Izumikawa, Richard J. Simpson, Nobuhiro Takahashi

**Affiliations:** 1 Department of Applied Biological Science, United-graduate School of Agriculture, Tokyo University of Agriculture and Technology, 3-5-8 Saiwai-cho, Fuchu, Tokyo 183-8509, Japan; 2 Core Research for Evolutional Science and Technology (CREST), Japan Science and Technology Agency (JST), Sanbancho 5, Chiyoda-ku, Tokyo 102-0075, Japan; 3 The Genome Science human resource development program, Tokyo University of Agriculture and Technology, 3-5-8 Saiwai-cho, Fuchu, Tokyo 183-8509, Japan; 4 Centre for Gene Regulation & Expression, College of Life Sciences, University of Dundee, Dow Street, Dundee, DD1 5EH, UK; 5 La Trobe Institute for Molecular Science (LIMS) LIMS Building 1, Room 412 La Trobe University, Bundoora Victoria 3086, Australia

## Abstract

Collaborator of alternative reading frame protein (CARF) associates directly with ARF, p53, and/or human double minute 2 protein (HDM2), a ubiquitin-protein ligase, without cofactors and regulates cell proliferation by forming a negative feedback loop. Although ARF, p53, and HDM2 also participate in the regulation of ribosome biogenesis, the involvement of CARF in this process remains unexplored. In this study, we demonstrate that CARF associates with 5′-3′ exoribonuclease 2 (XRN2), which plays a major role in both the maturation of rRNA and the degradation of a variety of discarded pre-rRNA species. We show that overexpression of CARF increases the localization of XRN2 in the nucleoplasm and a concomitant suppression of pre-rRNA processing that leads to accumulation of the 5′ extended from of 45S/47S pre-rRNA and 5′-01, A0-1 and E-2 fragments of pre-rRNA transcript in the nucleolus. This was also observed upon XRN2 knockdown. Knockdown of CARF increased the amount of XRN2 in the nucleolar fraction as determined by cell fractionation and by immnocytochemical analysis. These observations suggest that CARF regulates early steps of pre-rRNA processing during ribosome biogenesis by controlling spatial distribution of XRN2 between the nucleoplasm and nucleolus.

## INTRODUCTION

Alternative reading frame protein (ARF; known as p14^ARF^ in humans or p19^ARF^ in mice) is involved in the p53 tumor-suppressor pathway (1) in which ARF inhibits the ubiquitin–protein ligase Mdm2 (or HDM2), and leads to stabilization and elevated levels of p53 (2–5). ARF also suppresses growth of p53–Mdm2-deficient cells, suggesting that ARF can inhibit tumor growth independently of the p53 tumor-suppressor pathway (6). p53-independent tumor suppression by ARF is possibly due to the ability of ARF to suppress ribosome biogenesis by regulating the stability of B23 (7–14).

Collaborator of ARF (CARF), which was identified as an ARF-interacting protein based on yeast two-hybrid screening (15,16), is found ubiquitously in almost all human tissues. CARF is mainly localized in the nucleoplasm and co-localizes with ARF in the periphery (granular region) of nucleoli (15), where ribosome biogenesis takes place. Therefore, CARF may interfere with the role of ARF in ribosome biogenesis by interacting with ARF. CARF is involved not only in the ARF-dependent p53 pathway but also in the ARF-independent p53 pathway, both of which regulate tumor cell proliferation (15,17). In the ARF-dependent p53 pathway, CARF directly interacts with the ubiquitin–protein ligase Mdm2 in a complex with ARF (18,19) and thus cooperates with ARF in activating p53 (18). In the ARF-independent p53 pathway, CARF directly interacts with p53, stabilizing and functionally activating p53 (17); however, when the amounts of CARF and the p53 complex are elevated, these complexes are ubiquitinylated by the action of Mdm2 and subsequently proteolytically degraded (17). Thus, a feedback loop appears to exist in the CARF–p53 pathway in the absence of ARF, i.e. CARF activates p53, p53 activates Mdm2, and Mdm2 degrades CARF and p53 (15–19). In this feedback loop, CARF can also act as a transcriptional repressor of HDM2, the human counterpart of Mdm2 (19). An Mdm2 inhibitor interferes with this feedback network (20).

Overexpression of CARF induces premature senescence in human fibroblasts (21). Similarly, replicative and stress-induced senescence triggers an increase in CARF expression and activates the p53/p21^WAF^ (cyclin-dependent kinase inhibitor 1A) pathway (21). In contrast, CARF depletion induces apoptosis and abnormal cell division in cultured cells (21) and suppresses tumor growth in a human tumor xenograft mouse model (22). CARF depletion also affects various cell death and survival pathways, such as those involved in mitochondrial stress, ataxia telangiectasia mutated-ataxia telangiectasia and Rad3-related, Ras–mitogen-activated protein kinase, and retinoblastoma cascades (22). CARF is regulated by neuronal PAS domain protein 2 (NPAS2), a product of the circadian NPAS2 gene in MCF-10A cells (23). However, the molecular mechanisms by which CARF is involved in premature senescence, cell growth, and cell death remain unclear.

In this study, we examined CARF-interacting proteins using a proteomics approach to gain insight into the role of CARF. We show that CARF interact with 5′-3′ exoribonuclease 2 (XRN2) and may be implicated in the early steps of pre-rRNA processing.

## MATERIALS AND METHODS

### Construction of FLAG–CARF-expressing cell lines

A doxycycline-inducible FLAG–CARF-expressing cell line was established using the Flp-In T-Rex Expression System (Invitrogen, Carlsbad, CA). Briefly, Flp-In T-Rex 293 cells were cultured in one well of a 24-well plate (Thermo Fisher Scientific, Waltham, MA). At ∼50% confluency (visually estimated based on viewing through a microscope), they were transfected with 2 μl Lipofectamine 2000 (Invitrogen), 250 ng plasmid pcDNA5/FRT-TO-FLAG-CARF and pOG44 (Invitrogen). A clonal cell line was selected in normal medium; Dulbecco's modified Eagle's medium (Sigma-Aldrich, St. Louis, MO) [supplemented with 10% heat-inactivated fetal bovine serum (Biowest LLC, Miami, FL), 100 U/ml Penicillin G (WAKO Pure Chemicals, Osaka) and 100 μg/ml streptomycin sulfate (WAKO Pure Chemicals)], containing 100 μg/ml hygromycin B (Invitrogen) and named TOCARF cells. Other cell lines stably expressing HA–CARF (wt, N1, N2, C1, C2, and NC)–TEV–FLAG were established by transfecting Flp-In T-Rex 293 cells with 2 μl Lipofectamine 2000, 250 ng plasmid pcDNA5/FRT–HA–CARF (wt, N1, N2, C1, C2, and NC)–TEV–FLAG, and pOG44, followed by selection in normal medium containing 100 μg/ml hygromycin B. A cell line stably expressing FLAG–Fibrillarin was also established by transfecting Flp-In T-Rex 293 cells with 2 μl Lipofectamine 2000, 250 ng plasmid pcDNA5/FRT-FLAG-Fibrillarin and pOG44, followed by selection in normal medium containing 100 μg/ml hygromycin B.

### Immunoblot (IB) analysis

Proteins were separated with SDS–PAGE and electrophoretically transferred to a polyvinylidene difluoride membrane (85 mm × 55 mm; Millipore, Billerica, MA). The membranes were blocked with 5% non-fat dried skim milk in TBS for 1 h at 25°C and incubated with an appropriate primary antibody in 1% non-fat dried skim milk in TBS overnight at 4°C. After washing three times with TBS containing 0.1% (w/v) Tween 20 for 10 min, the membranes were incubated with a secondary antibody conjugated to alkaline phosphatase in 1% non-fat dried skim milk in TBS for 1 h, washed three times in TBS containing 0.1% (w/v) Tween 20 for 10 min, and washed once in TBS alone for 5 min. Membranes were stained in staining solution, which was prepared as a 1:50 dilution of nitro-blue tetrazolium chloride/5-bromo-4-chloro-3′-indolylphosphatase *p*-toluidine salt stock solution (Roche Diagnostics GmbH, Mannheim, Germany) in alkaline phosphatase buffer (100 mM Tris-HCl, pH 9.5, 100 mM NaCl, 50 mM MgCl_2_). Quantification of visualized protein bands was carried out using ImageJ software (http://rsbweb.nih.gov/ij/).

### Isolation of CARF-associated complexes

TOCARF cells were cultured to 80% confluency in four 150-mm cell culture dishes in normal medium with or without 1 ng/ml doxycycline for 48 h. After the cells were washed with phosphate buffer [PBS(−)], they were lysed in buffer A (50 mM Tris-HCl, pH 8.0; 150 mM NaCl; 0.5% IGEPAL CA-630; 1 mM Na_3_VO_4_; and 1 mM PMSF) and incubated on ice for 30 min. The cell extracts were obtained as the supernatant following centrifugation of the lysate at 4°C for 30 min at 22,180 × *g*. Cell extracts (15 mg) were incubated with anti-FLAG M2 agarose beads (Sigma) for 4 h at 4°C. The beads were then washed five times in buffer A and then once with buffer B (50 mM Tris-HCl, pH 8.0; 150 mM NaCl; 1 mM Na_3_VO_4_; and 1 mM PMSF). The FLAG–CARF-associated complexes were released from the anti-FLAG M2 agarose beads by adding 40 μl of 0.5 mg/ml FLAG peptide (Sigma) on ice for 30 min twice.

### In-gel protease digestion and liquid chromatography-tandem mass spectrometry (LC-MS/MS) analysis (GeLC-MS/MS)

These analyses were done by methods described by Fujiyama-Nakamura *et al*. (24) and Yanagida *et al*. (25), and described briefly in Supplementary Materials.

### Isolation of HA–XRN2-associated proteins

TOCARF cells were cultured to 25% confluency in two 90-mm cell culture dishes in normal medium with or without 100 ng/ml doxycycline. These cells were transfected with 10 μg pcDNA3.1 (+)-HA-XRN2 using the calcium phosphate method (26), cultured for 24 h, and incubated further in new medium for 24 h. After washing with Phosphate buffered saline (PBS), the cells were lysed in buffer A and incubated on ice for 30 min. Cell extracts were obtained as the supernatant following centrifugation of the lysate at 4°C for 30 min at 22,180 × *g*. The cell extracts from 1 × 10^7^ cells were incubated with 1 μg anti-HA and 15 μl protein G–Sepharose 4 Fast Flow beads (GE Healthcare) for 4 h at 4°C. The beads were washed six times in buffer A, and the HA–XRN2-associated complexes were released from the beads with 50 μl SDS sample buffer (50 mM Tris-HCl, pH 6.8; 2% SDS; 6% β-mercaptoethanol; 10% glycerol; and 0.05% bromophenol blue; BPB).

### Isolation of endogenous CARF- and XRN2-associated proteins

Antibody-conjugated affinity beads were incubated with 1 μg of normal rabbit IgG (Millipore), anti-CARF (Bethyl) or anti-XRN2 (Bethyl) with 15 μl Dynabeads protein G (Thermo) for 1 h at room temperature. 293T cells were cultured 90-mm cell culture dish in normal medium. After washing with PBS, the cells were lysed in buffer A on ice for 30 min and centrifuged at 22,180 × *g* at 4°C for 30 min. The supernatant (1 mg) was incubated with antibody-conjugated affinity beads for 3 h at 4°C. The beads were washed with buffer A 5 times, and the endogenous CARF or XRN2-associated proteins were released from the beads with 50 μl SDS sample buffer.

### Cell fractionation

After washing with PBS, the cells were lysed in 1 ml buffer C (16.7 mM Tris-HCl, pH 8.0; 50 mM NaCl; 1.67 mM MgCl_2_; 1 mM PMSF; 0.05% Triton X-100) and incubated on ice for 5 min. The cytosol was obtained as the supernatant following centrifugation of the lysate at 4°C for 5 min at 1,000 × *g*. The precipitate was washed with 1 ml buffer C, lysed in 0.5 ml buffer D (50 mM Tris-HCl, pH 8.0; 150 mM NaCl; 5 mM MgCl_2_; 1 mM PMSF), and sonicated twice for 20 s at an interval of 90 s with a Bioruptor (Cosmo Bio, Tokyo, Japan) at the highest setting. The nuclear extract fraction was obtained as the supernatant following centrifugation of this lysate at 4°C for 15 min at 15,000 × *g*. The pellet was lysed in 0.5 ml buffer D and sonicated 10 times for 20 s at intervals of 90 s with a Bioruptor at the highest setting. The pellet from centrifugation of this lysate at 4°C for 30 min at 15,000 × *g* was lysed in 0.5 ml buffer E (50 mM Tris-HCl, pH 8.0; 150 mM NaCl; 10 mM EDTA; 10 mM DTT; 1 mM PMSF) and sonicated 10 times as described above. The extract fraction representing the nucleolar/Cajal bodies was obtained as the supernatant following centrifugation of the latter lysate at 4°C for 30 min at 16,000&nbsp× *g*.

### Cell fractionation for immunoprecipitation (IP)

After washing with PBS, 1.2 × 10^7^ cells were lysed in 1 ml buffer F (16.7 mM Tris-HCl, pH 8.0; 50 mM NaCl; 1.67 mM MgCl_2_; 1 mM PMSF; and 0.1% Triton X-100) and incubated on ice for 3 min. The cytosol was obtained as the supernatant following centrifugation of the lysate at 4°C for 5 min at 1,000 × *g*. The precipitate was washed with 1 ml buffer F, lysed in 0.5 ml of 50 mM Tris-HCl, pH 8.0, containing 150 mM NaCl; 5 mM MgCl_2_; 1 mM PMSF; and 0.5% IGEPAL-CA630, and sonicated 10 times for 20 s at an interval of 90 s with a Bioruptor at the highest setting. Nuclear extract fraction-1 was obtained as the supernatant following centrifugation of this lysate at 4°C for 30 min at 16,000 × *g*. The precipitate was lysed in 0.5 ml buffer E and sonicated 10 times as described above. Nuclear extract fraction-2 was obtained as the supernatant following centrifugation of the latter lysate at 4°C for 30 min at 16,000 × *g*.

### Immunocytochemical staining

Collagen-coated culture slides were prepared by adding 100 μl of 50 μg/ml rat tail collagen type I (Becton, Dickinson and Company, Franklin Lakes, NJ), which was dissolved in 0.02 N acetic acid, into each well of an 8-well culture slide (Becton, Dickinson and Company). Cells were grown on collagen-coated culture slides that were washed with PBS. After the culture medium was removed and the slide was washed with PBS, the cells were fixed with 3.7% formaldehyde in PBS for 10 min at 25°C. The cells were washed with PBS containing 0.05% (w/v) Tween 20 (PBST), and permeabilized with PBS containing 0.5% (w/v) Triton X-100 for 5 min at 25°C. The cells were then blocked with 3% (w/v) non-fat dried skim milk in PBS for 1 h, incubated with the appropriate primary antibody for 1 h at 25°C, washed three times with PBST for 10 min, and incubated with a fluorochrome-conjugated secondary antibody for 1 h at 25°C. Finally, after being washed three times with PBST for 10 min, the cells were mounted using VECTASHIELD Mounting Medium with DAPI (Vector Laboratories, Burlingame, CA) and visualized with an Axiovert 200 M microscope (Carl Zeiss, Oberkochen, Germany).

### Detection of protein–protein interactions using the mKG reporter system

Protein–protein interactions were detected in cells using the mKG reporter system (MBL, Nagoya, Japan) according to the manufacturer's instructions. Flp-In T-Rex 293 cells were cultured in collagen-coated culture slides and at a confluency of ∼60% (visually estimated based on viewing through a microscope) were transfected with 1.5 μl Lipofectamine 2000 and 750 ng of the following plasmids: phmKGN–MC–FLAG–CARF and phmKGC–MC–XRN2, phmKGC-MC-FLAG-CARF and phmKGN-MC-XRN2, without plasmid as a negative control, or with pCONT-1 and pCONT-2 (MBL) as a positive control. The cells were fixed and permeabilized 48 h after transfection as described above and then were washed three times with PBST. The cells were mounted using VECTASHIELD Mounting Medium with DAPI and visualized with an Axiovert 200 M microscope. The cells were immunostained as described to detect transfected proteins. Imaging experiments using the mKG reporter system were repeated at least twice using independent transfections.

### Northern blot hybridization

About 293T cells were cultured until 70% confluency in 35-mm cell culture dish. Cells were transfected with scRNA (control) or siRNA for XRN2 knockdown using Lipofectamin RNAiMAX reagent (Invitrogen). After 24 h transfection, the cells were cultured in 100-mm cell culture dish for 48 h. TRex or TOCARF cells were cultured until 80% confluency in 100-mm cell culture dish in the presence (10 ng/ml) or absence of doxycycline for 72 h.

Total RNAs were isolated from the growing sub-confluent cells using the RNAgent Total RNA Isolation System (Promega, Madison, WI) followed by separation with acid phenol–chloroform extraction. The RNAs (2–2.5 μg) were electrophoresed in an agarose/formaldehyde gel and transferred to a Hybond N+ membrane (GE Healthcare). After staining with methylene blue, the membrane was hybridized to biotin-labeled probes at 50°C overnight in pre-hybridization solution: 5x SSC; 20 mM Na_2_HPO_4_, pH 7.2; 7% SDS; 2x Denhardt's Solution (0.02% [w/v] polyvinylpyrrolidone, Sigma-Aldrich; 0.02% [w/v] Ficoll, Sigma-Aldrich; 0.02% [w/v] bovine serum albumin, Sigma-Aldrich); and 1 mg/ml salmon sperm DNA. The membrane was washed with non-stringent wash solution (3x SSC; 25 mM NaH_2_PO_4_, pH 7.5; 5% SDS) once for 30 min at 50°C, and once with stringent wash solution (1x SSC, 1% SDS) for 30 min at 50°C. The hybridized RNA was detected using a Chemiluminescent Nucleic Acid Detection Module kit (Thermo Fisher Scientific). The following oligonucleotides were used as probes to detect human pre-rRNAs with northern blot hybridization: 5′ external transcribed spacer (5′ ETS)-1 probe (5′-TCGGACGCGCGAGAGAACAGCAGG-3′), complementary to nt 132 to 155 of the 5′ ETS; 5′ ETS-2 probe (5′-AGACGAGAACGCCTGACACGCACGGCAC-3′), complementary to nt 297 to 324 of the 5′ ETS; 5′ ETS-3 probe (5′-ACAGCGACGGAGGCAATACC-3′), complementary to nt 1468 to 1487 of the 5′ ETS; 5′ ETS-4 probe (5′-TCACGCGCCGGACAGAG-3′), complementary to nt 1731 to 1747 of the 5′ ETS. ITS1-1 probe (5′-GTCTTTAAACCTCCGCGCCGGAACGCGCTAGGTAC-3′) to nt 594 to 628 of the ITS1; 5.8S probe (5′- AGACAGGCGTAGCCCCGGGAGGAA -3′) to nt 123 to 146 of the 5.8S; ITS2-1 probe (5′- ACGCCGCCGGGTCTGCGCTTA-3′) to nt 93 to 113 of the ITS2. Probes were 3′-end labeled using a Biotin 3′-End DNA Labeling kit (Thermo Fisher Scientific).

## RESULTS AND DISCUSSION

### CARF associates with XRN2

**Figure 1.**
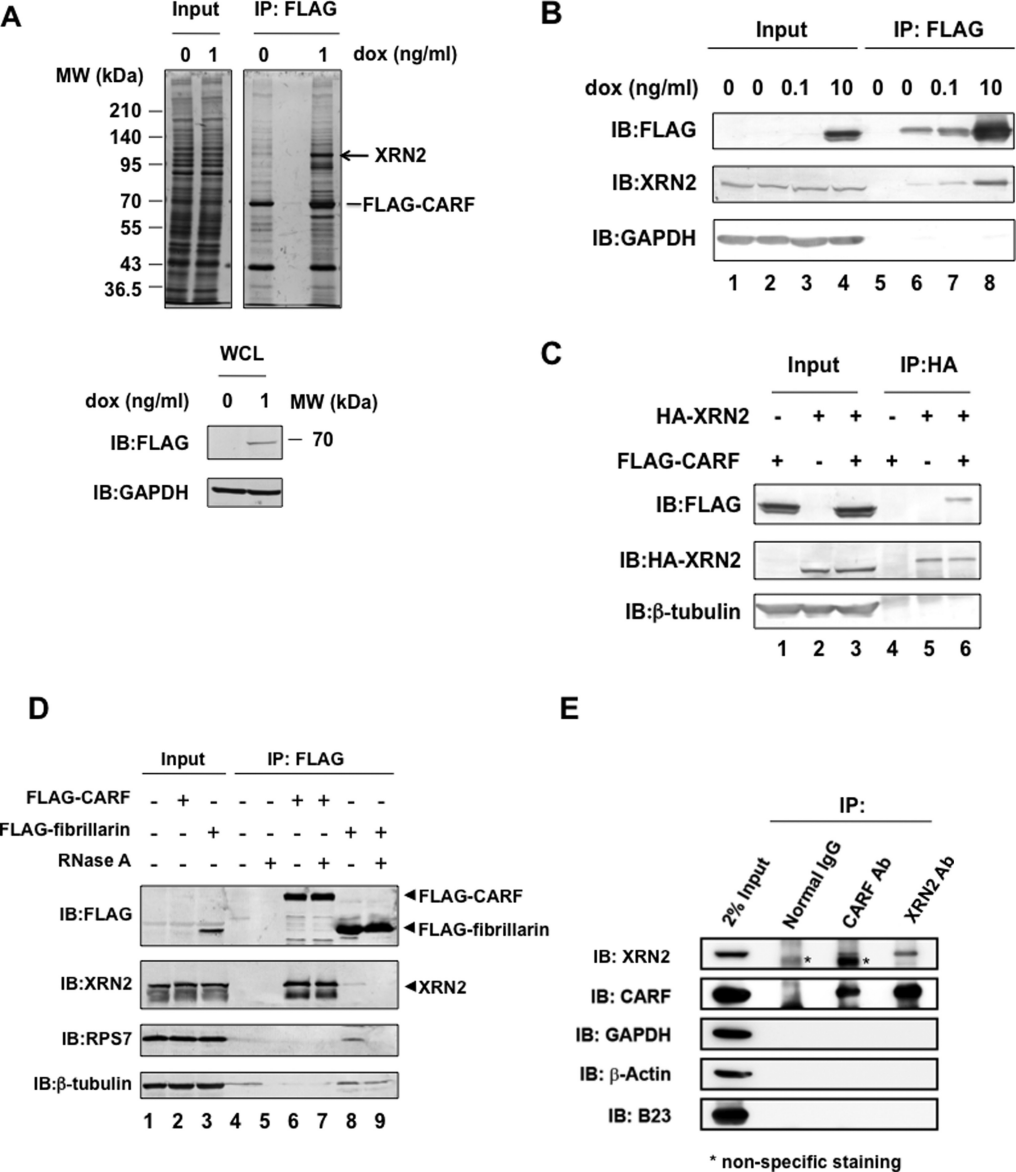
CARF associates with XRN2. (**A**) Using anti-FLAG, FLAG–CARF-associated complexes were immunoprecipitated from the extract of TOCARF cells after induction with 1 ng/ml doxycycline (dox). The FLAG–CARF-associated complexes (1 μg protein/lane) were separated on a 7.5% SDS-polyacrylamide gel and visualized with silver staining. Molecular weights (MW) are given on the left. Whole-cell lysate (WCL) were analyzed with immunoblotting with the indicated antibodies (20 μg protein/lane). (**B**) FLAG–CARF-associated complexes were immunoprecipitated with anti-FLAG from TOCARF cells after induction with doxycycline (lanes 2 and 6, 0 ng/ml; lanes 3 and 7, 0.1 ng/ml; lanes 4 and 8, 10 ng/ml), and the immunoprecipitates from Flp-In T-Rex 293 cells (lanes 1 and 5) were analyzed with immunoblotting with the antibodies indicated on the left (IB). The input fraction for each cell preparation (2 × 10^5^ cells/lane) was also analyzed. (**C**) HA–XRN2-associated complexes were immunoprecipitated from cells with anti-HA following treatment: induction of CARF expression with 100 ng/ml dox (lanes 1, 3, 4 and 6) and transfection with an HA–XRN2 expression plasmid (lanes 2, 3, 5 and 6). Input fractions (2 × 10^5^ cells/lane) and each complex were analyzed with immunoblotting using the antibodies indicated on the left. (**D**) Using anti-FLAG, FLAG–CARF-associated complexes were immunoprecipitated from cells that stably expressed FLAG–CARF. Immunoprecipitates were treated without (−) or with (+) RNase A (lanes 6 and 7). Controls were performed with Flp-In T-Rex 293 cells that were untransfected (lanes 4 and 5) and that stably expressed FLAG–fibrillarin (lanes 8 and 9). Input fractions (10 μg protein/lane) and each complex were analyzed with immunoblotting using the antibodies indicated on the left. (**E**) CARF- and XRN2-associated complexes were immunoprecipitated with anti-CARF and anti-XRN2 from 293T cell lysate (1 mg/1 ml), respectively (lanes 3 and 4). Normal rabbit IgG was used as a control (lane 2). Input fraction (2%) and complexes were analyzed with immunoblotting using the antibodies indicated on the left.

To gain insight into the role of CARF in cellular function, we examined proteins associated with CARF using a combination of an epitope-tagged pull-down methodology and LC-MS/MS (28). We used a site-directed (Flp-In) recombinase-based system to generate isogenic cell lines (27) in which a cytomegalovirus promoter was used to drive the expression of CARF that was integrated at a common locus in the Flp-In T-Rex 293 genome (28). A product of a single-copy transgene of *CARF* was tagged at the amino terminus with a FLAG affinity purification tag that is used for visualization and purification. Using this approach, we obtained isogenic cell lines expressing FLAG-tagged CARF (FLAG–CARF) with a molecular weight of ∼70 kDa, which corresponds to that estimated from its amino acid sequence. FLAG–CARF was localized mostly in the nucleoplasm (Supplementary Figure S1A) as reported (17). We analyzed FLAG–CARF-associated proteins from TOCARF cell lysate using anti-FLAG mAb coupled beads. Associated proteins were separated by SDS–PAGE, visualized by silver staining and several candidate proteins were identified by LC-MS/MS (28). Prominent amongst these was XRN2 (Mascot protein score 1.168, 35 peptides representing 36.5% sequence coverage, Supplementary Table S1). Immunoblot analysis with anti-XRN2 indicated that XRN2 corresponded to a protein band with a molecular weight of ∼100 kDa and most strongly stained among the proteins associated with FLAG–CARF on the SDS–PAGE gel (Figure 1A, arrow). Although mass-based analysis did not identify ARF, it does not necessarily mean that ARF is not there; in a complex mixture, ARF-related peptide ion signals may be masked/sequested by more abundant peptide ion signal, or ARF may be of very low abundance. We know minimally that the structural integrity of recombinant CARF overexpressed in Flp-In T-Rex 293 cells is correct, given that FLAG–CARF can interact with HA-tagged ARF when co-expressed in Flp-In T-Rex 293 cells (Supplementary Figure S1B) (15).

To further ascertain the interaction between CARF and XRN2, we performed an additional IP using TOCARF cells. FLAG–CARF binding to XRN2 was increased in a doxycycline dose-dependent manner (Figure 1B). In addition, we performed reverse pull-down analysis using HA-tagged XRN2 (HA–XRN2) as bait and observed a reciprocal interaction (Figure 1C). Because CARF has a putative double-stranded RNA-binding domain (http://www.ebi.ac.uk/interpro/protein/Q9NXV6) and XRN2 is the homolog of yeast XRN2/Rat1, which processes RNAs with its 5′-3′ exoribonuclease activity (29–32), we tested whether they associated with each other in the presence of RNA. We treated the immunoprecipitate obtained from the Flp-In T-Rex 293 cells that stably expressed FLAG–CARF with RNase A and observed that XRN2 remained associated with CARF even after RNase treatment (Figure 1D), indicating that CARF associates with XRN2 independently of RNA. The RNase treatment released ribosomal protein S7 from fibrillarin, confirming the RNase activity. To rule out the possibility that the interactions were due to overexpression of HA–XRN2, we next immunoprecipitated endogenous XRN2 from 293T cells using an antibody against endogenous XRN2 and showed the interaction between the two proteins (Figure 1E). We also immunoprecipitated endogenous CARF from 293T cells using an antibody against endogenous CARF, and showed reciprocal interaction between the two proteins (Figure 1E).

### CARF binds directly to XRN2 in the nucleoplasm

**Figure 2.**
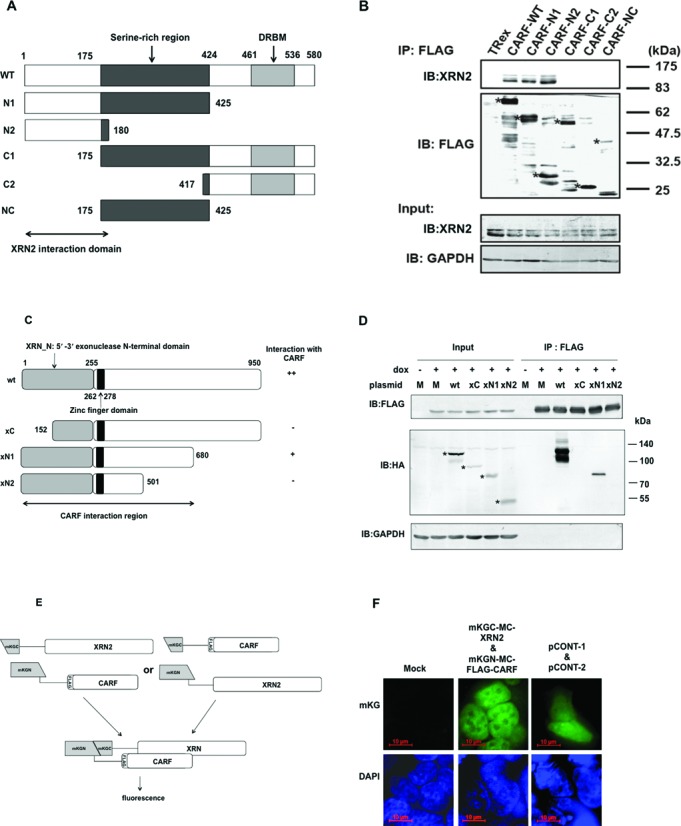

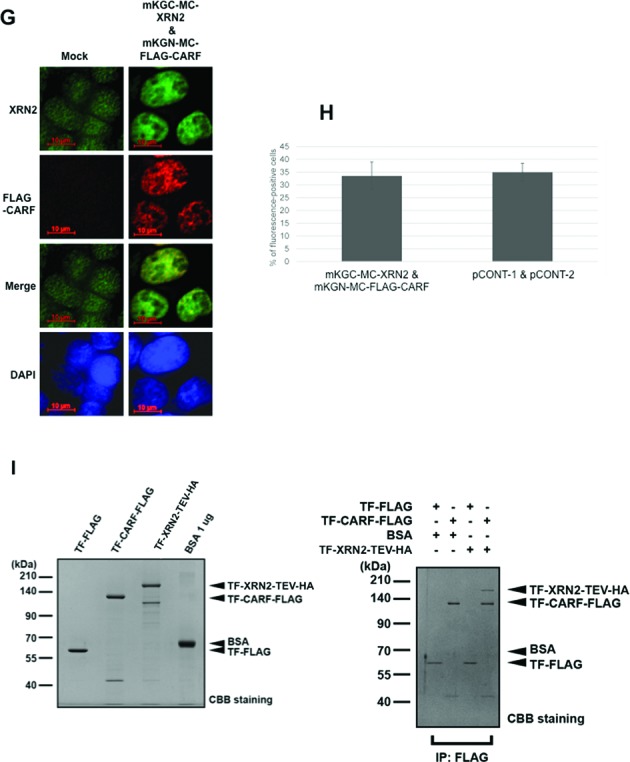
CARF interacts directly with XRN2. (**A**) Schematic representation of CARF deletion mutants and the CARF-XRN2 interaction region in CARF. DRBM; double strand RNA-binding domain. (**B**) FLAG–CARF-associated complexes were immunoprecipitated with anti-FLAG from Flp-In T-Rex 293 cells that were transfected with FLAG–CARF mutants and empty vector as control (TRex). These immunoprecipitates were analyzed with immunoblotting using the antibodies indicated on the left. The input fraction for each cell preparation (30 μg per lane) was also analyzed and detected with the antibodies indicated on the left. The molecular sizes of molecular standards are indicated at the corresponding mobilities at right. (**C**) Schematic representation of XRN2 deletion mutants and CARF–XRN2 interaction region in XRN2. (**D**) FLAG–CARF-associated complexes were immunoprecipitated using anti-FLAG from TOCARF cells that were transfected with HA–XRN2 mutants or empty vector as control (M: Mock) after induction with (+) or without (−) doxycycline (dox) (10 ng/ml) for 72 h. These immunoprecipitates were analyzed with immunoblotting using the antibodies indicated on the left. Input fraction of each cell preparation (20 μg per lane) was also analyzed. (**E**) Schematic representation of the detection of protein–protein interactions by fluorescence microscopy using the mKG reporter system in cells. mKG fluorescence was reconstituted in transfected cells when fused to interacting N-terminal mKG fragment (mKGN)-tagged FLAG–CARF and C-terminal mKG fragment (mKGC)-tagged XRN2, or *vice versa* (data not shown). (**F**) The interaction between FLAG–CARF and XRN2 was detected with mKG fluorescence (green) in the second panel. A combination of mKGC–MC–XRN2 and mKGN–MC–FLAG–CARF (second panel) was examined. Flp-In T-Rex 293 cells were transfected with pCONT-1 and pCONT-2 as positive controls on the right. DAPI: corresponding images (bottom) were stained for double-stranded DNA to indicate the nuclei. Scale bars, 10 μm. (**G**) Flp-In T-Rex 293 cells that were transfected with mKGN-tagged FLAG–CARF and (mKGC)-tagged XRN2, or with no plasmid (as a control) were immunostained using an anti-XRN2 (green) or anti-FLAG (red). Scale bars, 10 μm. (**H**) Percent of mKG fluorescence-positive cells/DAPI-positive cells. The transfection efficiency of phmKGN–MC–FLAG–CARF was calculated using the ratio of FLAG–CARF-positive cells/DAPI-positive cells. Data were obtained from duplicate experiments. (**I**) The isolated recombinant FLAG–CARF (TF–CARF–FLAG) and HA–XRN2 (TF–XRN2–TEV–HA), and bovine serum albumin (BSA) were separated by SDS–PAGE and visualized by CBB staining (left). Binding assay was executed by mixing the recombinant proteins and following pulldown analysis with anti-FLAG conjugated agarose resin (right).

To identify the region in CARF that was responsible for binding to XRN2, we first constructed expression vectors for five FLAG-tagged truncated CARF mutants (N1, N2, C1, C2 and NC) (Figure 2A). These mutants were constructed based on the exon-intron boundaries of *CARF* (http://www.ncbi.nlm.nih.gov/nuccore/NM_017632.2) and the domain structure of human CARF (http://www.ncbi.nlm.nih.gov/protein/NP_060102.1). The expression and cellular localization of the truncated proteins were examined with SDS–PAGE and immunocytochemistry, respectively (Figure 2B, asterisks). All truncated CARF mutants were expressed in the cells and showed the expected molecular sizes as estimated from their amino acid sequences (Figure 2B). IB with anti-XRN2 showed that domain mutants N1 and N2 were associated with XRN2 (Figure 2B). Those two mutants were localized mainly in the nucleoplasm, similar to wild-type CARF (Supplementary Figure S2A), suggesting that the interaction between CARF and XRN2 occurs in the nucleoplasm. In contrast, all other mutants lacking the amino-terminal region corresponding to amino acids 1–175 did not associate with XRN2 (Figure 2B). Because those mutants were not localized in the nucleoplasm (Supplementary Figure S2A), we constructed additional expression vectors for C1 and C2 mutants fused to nuclear localization signal (NLS) and confirmed their nuclear localization (Supplementary Figure S2B). Those mutants, however, did not associate with XRN2 (Supplementary Figure S2C). Thus, N-terminal region of CARF (amino acids 1–175) is critical for its interaction with XRN2. It is interesting that mutant CARFs (N1 and N2), which can bind XRN2, seem to keep the ability to localize XRN2 in the nucleoplasm (Supplementary Figure S2A).

We next constructed expression vectors for three HA-tagged truncated XRN2 mutants (xC, xN1 and xN2) to identify the region in XRN2 that was responsible for binding to CARF (Figure 2C). These truncated mutants were constructed based on the exon-intron boundaries of *XRN2* (http://www.ncbi.nlm.nih.gov/nuccore/NM_012255.3) and the domain structure of human XRN2 (http://www.ncbi.nlm.nih.gov/protein/NP_036387.2). All truncated mutants were localized in the nucleoplasm of their expressing cells (Supplementary Figure S2D) and showed the expected molecular sizes as estimated from their amino acid sequences (Figure 2D, asterisks). IB with anti-HA showed that domain mutant xN1 was associated with CARF (Figure 2D). Thus, the minimal region of XRN2 corresponding to xN1 (amino acids 1–680) is necessary for the interaction with CARF.

In addition, we used the mKG reporter system to detect an *in vivo* interaction between XRN2 and CARF in Flp-In T-Rex 293 cells. Flp-In T-Rex 293 cells were transfected with a combination of phmKGN–MC–FLAG–CARF and phmKGC–MC–XRN2 vectors, two empty vectors as a negative control, or pCONT-1 and pCONT-2 (MBL) as a positive control. In this system, mKG fluorescence is reconstituted in transfected cells when CARF and XRN2 interact with each other (Figure 2E). Fluorescence was detected in the nucleoplasm of Flp-In T-Rex 293 cells that were transfected with phmKGN–MC–FLAG–CARF and phmKGC–MC–XRN2 vectors, but not with empty vectors (Figure 2F). The expression of FLAG–CARF and XRN2 in the Flp-In T-Rex 293 cells that were transfected with phmKGN–MC–FLAG–CARF and phmKGC–MC–XRN2 vectors was confirmed with immunocytochemistry using anti-FLAG and anti-XRN2 antibodies (Figure 2G). The transfection efficiency of phmKGN–MC–FLAG–CARF and phmKGC–MC–XRN2 vectors was about 30.9% as calculated by ratio of the number of FLAG–CARF-positive cells versus that of DAPI-positive cells (data not shown). This efficiency was very similar to that obtained using Flp-In T-Rex 293 cells transfected with the positive control pCONT-1 and pCONT-2 vectors (Figure 2H). Finally, we prepared recombinant CARF–FLAG and showed its interaction with XRN2 (Figure 2I). Coupled with the report that human XRN2 interacts directly with CARF (33), these data suggest that CARF interacted with XRN2 in the nucleoplasm and that the binding does not require cofactors.

### CARF suppresses early steps of pre-rRNA processing and the degradation of 5′-ETS fragments

**Figure 3.**
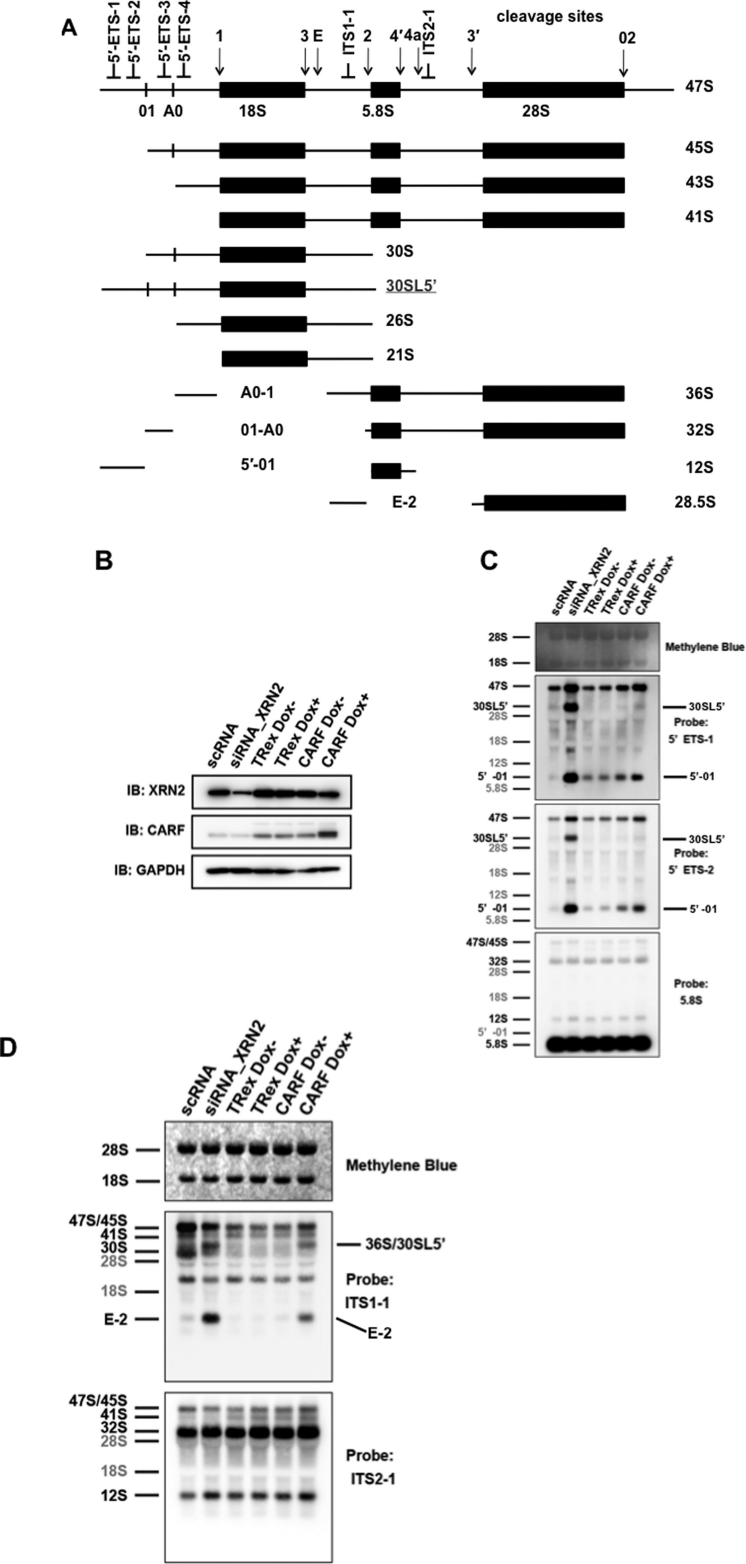
CARF suppresses the function of XRN2 in pre-rRNA processing. (**A**) Localization of the DNA probes within the human ribosomal transcription unit and a schematic of pre-rRNAs detected with hybridization. Aberrant pre-rRNAs lacking 01 cleavage are underlined and denoted in gray. (**B**) Whole-cell extracts from TOCARF or TRex cells after treating with (Dox+) or without (Dox−) 10 ng/ml doxycycline for 72 h (10 μg per lane) or after treating with scRNA or siRNA for XRN2 were analyzed with immunoblotting using the indicated antibodies on the left. (**C**) Northern blotting analysis of pre-rRNA isolated from the cells indicated in (**B**). Membranes were hybridized with the oligonucleotide probes (5′ETS1, 5′ETS2, 5.8S, see **A**). The pre-rRNAs and processed fragments are indicated at left based on the sizes and the specificity to the probes used. (**D**) Membranes were hybridized with oligonucleotide probes (ITS1-1, ITS2-1 see **A**).

XRN2 plays a major role in both the maturation of rRNA and the degradation of the 5′-extended form of 34.5S- and 45.5S-pre-rRNAs, and in the degradation of 5′-A′ and 19S segments, and it has also a role in degradation of the E-2 fragment, which is generated by two endonucleolytic cleavages at site E and site 2 in the ITS1 region in mouse and human cells (34–36). Thus, we postulated that CARF plays a role in pre-rRNA processing by interacting with XRN2. To test this idea, we first examined the effects of transient overexpression of CARF on the processing of 5′-ETS regions of pre-rRNA. We used four probes, 5′-ETS-1, 5′-ETS-2, 5′-ETS-3 and 5′-ETS-4, to detect the 5′-ETS region with northern blotting (Figure 3A). Overexpression of FLAG–CARF resulted in the accumulation of 5′-01 fragments (detected only with 5′-ETS-1 and 5′-ETS-2), A0-1 fragments (detected only with 5′-ETS-4), and 47S (detected with 5′-ETS-1 and 5′-ETS-2) relative to 28S rRNA methylene blue staining as compared with controls that expressed only the FLAG epitope (Supplementary Figure S3A–S3D). Consistent with these results, overexpression of FLAG–CARF significantly reduced 43S/45S/47S pre-rRNA detected with 5′-ETS-4, whereas it did not affect the processing of 30S pre-rRNA detected with 5′-ETS-3 and -4 (Supplementary Figure S3B). Although 30SL5′ pre-rRNA was only faintly detected with 5′-ETS-2 and was not significantly increased upon the transient expression of FLAG–CARF (Supplementary Figure S3A), we detected significant increase of 30SL5′ pre-rRNA with 5′-ETS-1 and 5′-ETS-2 upon the increased expression of FLAG–CARF in Flp-In T-Rex cells treated with doxycycline for 72 h (Figure 3C). In addition, we observed that the increased expression of FLAG–CARF resulted in the accumulation of 5′-01, and 45S/47S pre-rRNA (Figure 3B–C). We also used two additional probes (ITS1-1 and ITS2-1) to detect ITS1 and ITS2 regions by northern blotting, respectively (Figure 3A), and showed that overexpression of FLAG–CARF also accumulated 36S/30SL5′ and E-2 fragment (Figure 3D). Thus, CARF suppresses the degradation of 5′-01, A0-1, and the 5′-extended form of 45S-pre-rRNA as well as the degradation of E-2; thus, it also affects not only the processing of 5′ETS but also the processing of ITS1. These effects of overexpression of FLAG–CARF on the processing of pre-rRNA were very similar to those obtained by knockdown of XRN2 with stealth siRNAs in HeLa cells (Figures 3B–D), though we did not detect the processing of the 5′end maturation of the 28S rRNA that was also accumulated by the knockdown of XRN2 (34). Thus, CARF plays a role in suppressing the action of XRN2 during the processing of pre-rRNA.

### CARF retains XRN2 in the nucleoplasm

To gain insight into the mechanism by which CARF suppresses the action of XRN2 during the early processing of pre-rRNA, we first examined whether CARF directly inhibits the nucleolytic activity of XRN2 by interacting with XRN2. We constructed expression vectors encoding FLAG–TEV–HA-tagged XRN2 (WT) and FLAG–TEV–HA-tagged XRN2 lacking the amino-terminal exonuclease domain (ΔN278) and transiently expressed them in Flp-In T-Rex 293 cells. WT and ΔN278 fragments were prepared by pull-down using anti-FLAG-fixed beads (Supplementary Figure S4A). The WT protein showed nuclease activity when mixed with the synthetic RNA substrate (pSTP19 fragment), whereas the ΔN278 protein did not (Supplementary Figure S4B). We then added recombinant GST–FLAG–CARF (Supplementary Figure S4C) to this assay system, but we found no evidence that CARF affected the RNase activity of XRN2 (Supplementary Figure S4B). Although the construct was different, minimally the recombinant TF–CARF was able to bind to XRN2 (Figure 2I). We next examined whether knockdown of CARF affects the expression level of endogenous XRN2 or *XRN2* mRNA in cells. We observed no change in the expression level of XRN2 or *XRN2* mRNA with knockdown of CARF as examined with IB using anti-XRN2 (Supplementary Figure S4D). We also observed no change in the expression level of endogenous XRN2 or *XRN2* mRNA upon doxycycline-induced overexpression of FLAG–CARF (Supplementary Figure S4E).

**Figure 4.**
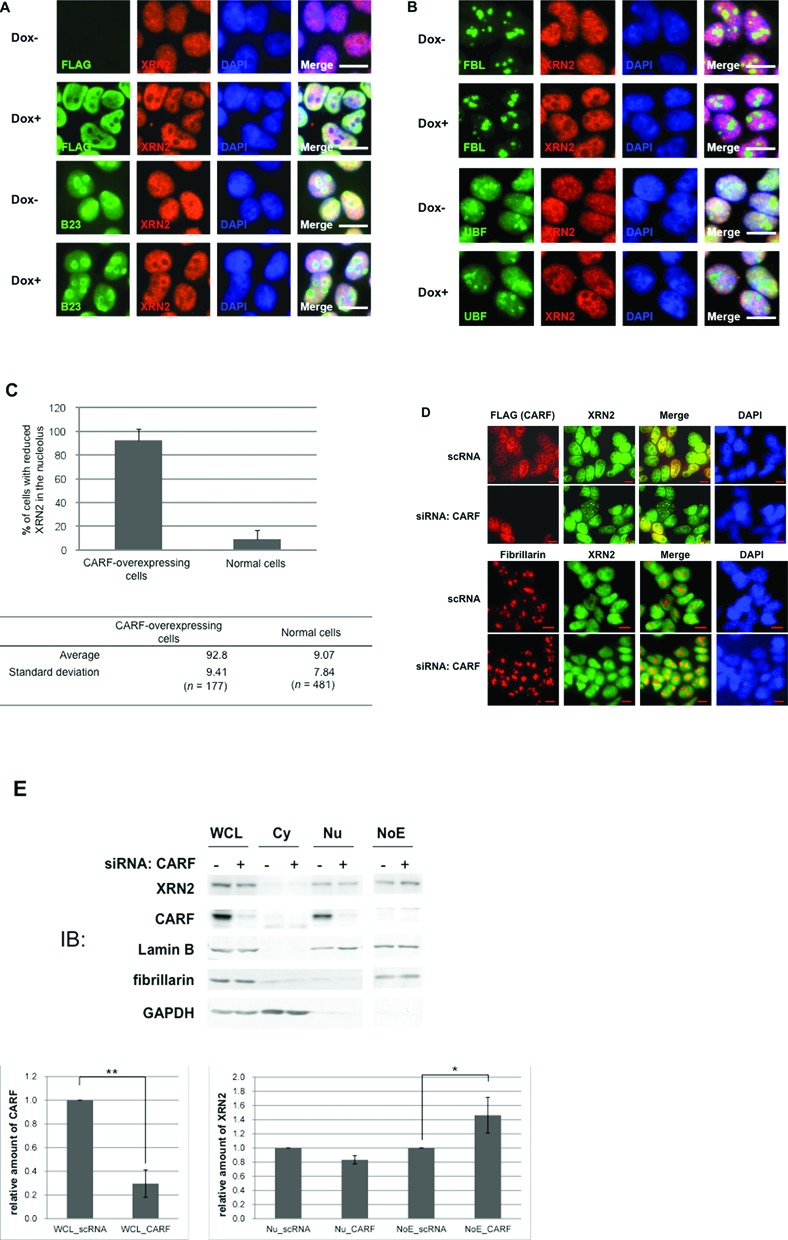

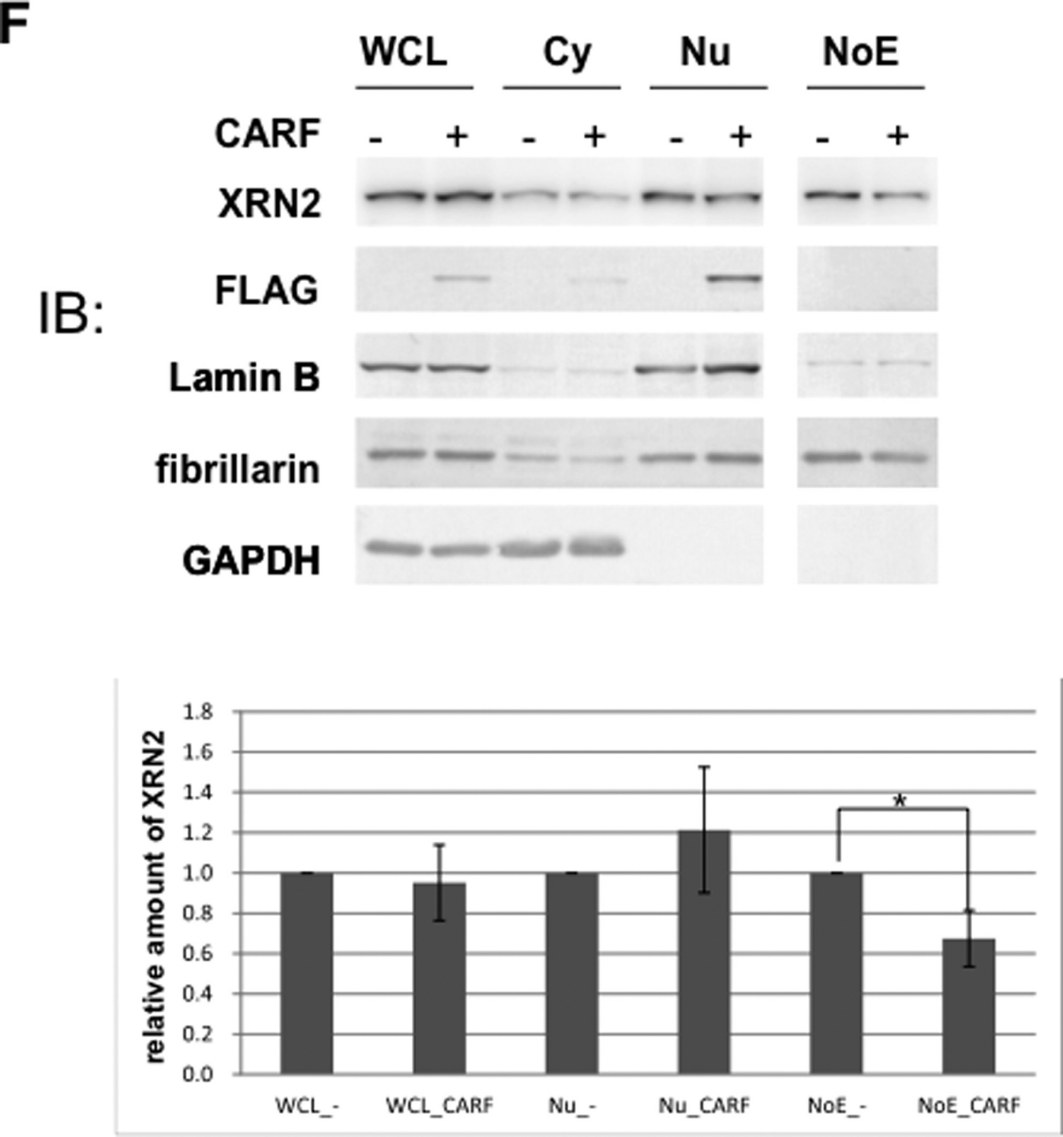
CARF retains XRN2 in the nucleoplasm. (**A**) TOCARF cells were treated without (Dox−) and with (Dox+) doxycycline, and were immunostained using an antibody against FLAG (green) and XRN2 (red). Those cells were also immunostained using an antibody against B23 (green) and XRN2 (red) in lower part. DAPI, corresponding images stained for double-stranded DNA indicating the nuclei. Scale bars, 10 μm. (**B**) Flp-In T-Rex 293 cells were immunostained using anti-fibrillarin (FBL) (green) and anti-XRN2 (red) (top two). These cells were also immunostained using anti-UBF (green) and anti-XRN2 (red) (bottom two). DAPI: corresponding images were stained for double-stranded DNA to indicate the nuclei. Scale bars, 10 μm. (**C**) Percentage of cells without XRN2 in the nucleolus in CARF-overexpressing cells or in cells expressing normal levels of CARF. Statistical significance was evaluated using the Student's *t*-test. *P* < 0.01. (**D**) Flp-In T-Rex 293 cells stably expressing HA–CARF–TEV FLAG were transfected with siRNA targeting CARF (bottom) or control scRNA (top) and were immunostained using anti-XRN2 (green) or anti-FLAG (red). These cells were also immunostained with anti-fibrillarin (red) (bottom two). DAPI: corresponding images stained for double-stranded DNA to indicate the nuclei. Scale bars, 10 μm. (**E**) Cells that were transfected with scRNA (−) or siRNA targeting CARF (+) were fractionated into cytosolic (Cy) and the nuclear extracts (Nu), the latter of which was used to prepare the nucleolar fraction (NoE) using cell fractionation. These fractions and WCL (10 μg protein per lane) were analyzed with immunoblotting using the antibodies indicated on the left. The proportion of XRN2 staining intensity of siRNA lane to total protein amount per lane was also normalized to that of scRNA lane. The results of immunoblotting were quantified and shown in a graph. Mean values (±SD) from three independent experiments are shown. *P < 0.05; **P < 0.01. (**F**) Flp-In T-Rex 293 cells stably expressing HA–CARF–TEV FLAG or Flp-In T-Rex 293 cells (−) were fractionated and analyzed as in **E**. The proportion of XRN2 staining intensity of FLAG–CARF (+) lane to total protein amount per lane was normalized to that of Flp-In T-Rex 293 cells (−) lane.

We finally considered the possibility that CARF affects the localization of XRN2 in the cell. Thus, we examined the cellular localization of XRN2 with immunocytochemistry before and after induction of FLAG–CARF in TOCARF cells. In the absence of induction of FLAG–CARF, XRN2 was dispersed throughout the nucleus without a clear boundary of its staining between the nucleoplasm and the nucleolus as shown by co-localization images of XRN2 with nucleolar markers specific for the 3 sub-region of the nucleolus in TOCARF cells (Figure 4A and B). In contrast, induction of FLAG–CARF reduced the nucleolar staining of XRN2 and revealed a clear boundary of XRN2 staining between the nucleoplasm and nucleolus (Figure 4A–C). We treated Flp-In T-Rex 293 cells expressing FLAG–CARF with stealth siRNA for CARF and observed dispersed localization of XRN2 in the nucleus of the siRNA-treated cells when compared with the scRNA treated cells expressing FLAG–CARF (Figure 4D). To assess the ability of CARF to further affect the localization of XRN2 in the nucleolus, we prepared the nucleolar fraction (NoE) from the nuclear extract of siRNA- or scRNA-treated HeLa cells using cell fractionation and analyzed the fractions with IB using anti-CARF and anti-XRN2. Knockdown with siRNA reduced CARF by 70% as compared with its level in the scRNA-treated cells and increased the proportion of XRN2 in the NoE fraction when compared with that prepared from the scRNA-treated cells (Figure 4E). The total amount of XRN2 did not differ between scRNA- and siRNA-treated cells. Conversely, overexpression of FLAG–CARF reduced the proportion of XRN2 in the NoE fraction (Figure 4F). Thus, CARF can affect the localization of XRN2 in the nucleolus.

In this study, we showed that XRN2 is another protein that binds directly to CARF, in addition to ARF, p53 and HDM2. XRN2 is a member of the eukaryotic 5PX family of exonucleases (37) that carry out 5′ to 3′ degradation of 5′-monophosphate-terminating RNA substrates and that are inhibited by a 5′-triphosphate or secondary structures in RNA (30,38). XRN2 is involved in pre-rRNA processing (34,35), and we also showed that CARF suppresses the processing of 45S/47S pre-rRNA and the degradation of 5′-01, A0-1 and E-2 fragments via its ability to retain XRN2 in the nucleoplasm. The present data demonstrate that CARF participates in pre-rRNA processing independently of its action on ARF, which regulates the stability of the rRNA-processing factor B23. Our observations confirmed the role of XRN2 in the processing of pre-rRNA and the degradation of its discarded fragments as reported by Wang & Pestov (34) and by others (35,36) and provided more details regarding the regulatory mechanism underlying mammalian pre-rRNA maturation and decay. CARF is expressed in all tissues (including brain, kidney, liver, lung, pancreas, placenta, colon and ovary) and in all cell lines (including U2OS, Saos-2, HeLa, C33A, H1299, WI38, WET, MRC5) tested so far, except for the cell line MCF7, and its expression level varies greatly among tissues and cell lines (16,17). The ubiquitous presence of CARF is consistent with its role in ribosome biogenesis, a fundamental function of the cell. Because the expression level of CARF regulates the availability of XRN2 in the nucleolus, different expression levels of CARF are expected to differently affect pre-rRNA processing in different tissues and cell lines. Thus, we have identified a new role for CARF in the regulation of the early processing of pre-rRNA and, possibly, in the regulation of cell growth.

Previous evidence indicates that CARF has a role in both cell growth and cell arrest. In addition, CARF overexpression results in growth arrest with no apparent signs of apoptosis in a cellular background lacking ARF, suggesting that overexpression of CARF supports p53-mediated growth arrest of cells independently of ARF (15). Our results provide another possible explanation for growth arrest, in which overexpressed CARF retains XRN2 in the nucleoplasm and suppresses the early steps of pre-rRNA processing. Perturbation of ribosome biogenesis activates p53, which leads to cell cycle arrest and apoptosis as reported in animal models of Treacher–Collins syndrome, a congenital disorder of craniofacial development arising from mutations in *TCOF1* (39,40). Therefore, overexpression of CARF may perturb ribosome biogenesis, leading to p53-dependent cell cycle arrest. CARF interacts with p53 in the nucleoplasm, stabilizing and functionally activating p53 in the absence of ARF (15,17). Thus, determining whether XRN2 competes with p53 to interact with CARF in the nucleoplasm will be interesting. An additional feedback loop may regulate cell growth and arrest.

Finally, in yeast, XRN2 is required for efficient termination by RNA polymerase I on the rDNA in cooperation with its cofactor Rai1 (32). Thus, CARF may also affect the termination of rDNA transcription. XRN2 is associated with lung cancer (41), and *XRN2* transcription is repressed by hypermethylation in adenoid cystic carcinoma (42). Thus, the association of CARF with XRN2 may have therapeutic potential as a novel tumor-suppressing target of anti-cancer reagents.

It is interesting to note the recent finding by Watanabe *et al*. that in HeLa cells mTOR regulates the diffusion of XRN2 from the nucleolus to the nucleoplasm under heat stress conditions and that this, in turn, may affect translational suppression through mTOR-regulated iMet degradation (43). It is interesting to speculate that heat stress and/or mTOR signaling affects the expression levels of nuclear CARF and XRN2 redistribution. Based on our present results, we suppose that this redistribution causes the suppression of early pre-rRNA processing. At the same time, it is also interesting to know whether ARF is diffused from the nucleolus to the nucleoplasm under the condition of heat stress. If ARF was diffused to the nucleoplasm, it is likely that ARF–CARF and CARF–XRN2 interactions occur simultaneously in the nucleoplasm. It will be very important to know whether the ARF–CARF interaction preclude CARF–XRN2 interaction in terms of negative feedback network among ARF, p53, Mdm2 and CARF as mentioned in INTRODUCTION. Clearly, a better understanding of CARF/XRN2 interactions and how XRN2 redistribution in the nucleus impacts on pre-rRNA processing may have far reaching therapeutic potential leading to novel tumor-suppressing targets.

During revision of this manuscript, Miki *et al*. reported that PAXT-1, a *C. elegans* homolog of human CARF, bound and stabilized XRN2 (33). Consistent with our present result, the binding did not alter the enzymatic activity of XRN2. In *C. elegans*, however, the two proteins regulate mutually their cellular levels, i.e. the elevated level of PAXT-1 stabilizes XRN2 and promotes its nuclease activity that is required for efficient degradation of miRNAs, whereas the reduced level of XRN2 decreases that of PAXT-1. These functions seem to be essential for the development of *C. elegans* (33). On the contrary, we did not observe the effect of the overexpressed CARF on stabilization of XRN2 in human cells we used so far. We are not sure at present whether those differences are species specific, cell specific or developmental stage specific ones. Since CARF participates in negative feedback loops through interaction with the other proteins including Mdm2, p53 and ARF in human cells, it is likely that the cellular levels of human CARF and XRN2 are regulated differently from those of *C. elegans*. Since XRN2 shows the nuclease activity on a number of different substrates, our present study possibly provides a mechanism by which CARF regulates the nuclease activity of XRN2 in substrate specific manner.

## Supplementary Material

SUPPLEMENTARY DATA
