# Supplementary material for: Collaborator of alternative reading frame protein (CARF) regulates early processing of pre-ribosomal RNA by retaining XRN2 (5′-3′ exoribonuclease) in the nucleoplasm
Source: Nucleic Acids Res. 2015 Nov 3;43(21):10397–410. doi: 10.1093/nar/gkv1069 (PMC4666357; doi:10.1093/nar/gkv1069)
Supplement: SUPPLEMENTARY DATA [file supp_43_21_10397__index.html]

Collaborator of alternative reading frame protein (CARF) regulates early processing of pre-ribosomal RNA by retaining XRN2 (5′-3′ exoribonuclease) in the nucleoplasm — SUPPLEMENTARY DATA 

# Collaborator of alternative reading frame protein (CARF) regulates early processing of pre-ribosomal RNA by retaining XRN2 (5′-3′ exoribonuclease) in the nucleoplasm

## SUPPLEMENTARY DATA

- SUPPLEMENTARY DATA
- SUPPLEMENTARY DATA
- SUPPLEMENTARY DATA
- SUPPLEMENTARY DATA
- SUPPLEMENTARY DATA
- SUPPLEMENTARY DATA
- SUPPLEMENTARY DATA
