# Supplementary material for: Collaborator of alternative reading frame protein (CARF) regulates early processing of pre-ribosomal RNA by retaining XRN2 (5′-3′ exoribonuclease) in the nucleoplasm
Source: Nucleic Acids Res. 2015 Nov 3;43(21):10397–410. doi: 10.1093/nar/gkv1069 (PMC4666357; doi:10.1093/nar/gkv1069)
Supplement: SUPPLEMENTARY DATA [file supp_gkv1069_Supplementary_Table_1.docx]

**Primer sequences**

**Allele –specific ChIP analysis**

| Primer | sequence |
| --- | --- |
| BlcapRTF | CTTTGGGTGGGAGAGGGC |
| BlcapRTR | CGGGCGGGGACAAATAGTA |
| Hdac4RTF | AGTGCCAATTACTGCTCTGGA |
| Hdac4RTR | GCCTGCGTCTCTCTGTAGTT |
| Oxct1RTF | TGAGTACCTCCTATGACCTCGT |
| Oxct1RTR | GCAGAGCCTTTCCCAGTTTG |
| TubeRTF | TAGGCCCTGCCTAGTGAG |
| TubeRTR | AAGCAACAGGAAGAAAGGACC |
| Trim3RTF | TCAAAGACGGCCTCACTACC |
| Trim3RTR | GGTAGGCATCTTCTGGGGAC |
| Ube2wRTF | AAACTCACATCACCAGGCCT |
| Ube2wRTR | TCTGGAGTAGAGTACGGAGCT |

**Methylation analysis of endogenous loci**

| **Primer** | **Sequence** |
| --- | --- |
| Tube1_BisF | TTGAATGTTATTTGAAATAGTTGAGT |
| Tube1_BisR | AAATCAAATAACACAAACAATTCAT |
| NhlrcZBF1 | TTATTTGTTTTTATGATGATGGTTA |
| NhlrcZBR1 | CATTTAAATAATATTAATATCTATTAACCC |
| Ube2ZBF1 | GGGATAGTTTTTTGAAGTTGTTTT |
| Ube2ZBR1 | AAATAAACTAACTTCCCATTACATCC |

**Gene transfer experiments**

| Primer | Sequence |
| --- | --- |
| Snrpn1For1 | AACACAGCTCTGACTTCCAGGAGTC |
| Snrpn1Rev1 | AATGTGCGCATGTGCAGCCATTGC |
| Snrpn1for2 | TGGAGATCTAACACAGCTCTGACTTCCAG |
| Snrpn1rev3 | CAGGTATACAATGTGCGCATGTGCAGCC |
| Mut1for2 | TGCTGAGGCAACACAGCTCCTGCATCC |
| Mut1rev2 | TTGCCTCAGCATGGGGGCTCCAGG |
| Mut2for2 | CAGGTGCAGCACTAACACACCCAAGGAG |
| Mut2rev2 | TTAGTGCTGCACCTGCGTGTAACGCG |
| Mut3for2 | CTTGCAGCAATGGCTCAGGTTTGTCGCG |
| Mut3rev2 | CATTGCTGCAAGACTAGCGCAGAGAGG |
| pCGfBmcsF | GTATATAAGGTTAATGGGAGGTAAGTTAATGGG |
| pCGfBmcsR | TCATATCTAACCAACTAACTCCATAATAAACCC |
| PCG1-ChIP-for | CAATGGGAGGTAAGCCAATG |
| PCG1-ChIP-rev | CTCCTCAGAACCAAGCGTCT |
| PCG2-ChIP-for | ATTGGTGAGCAATCCTTTGG |
| PCG2-ChIP-rev | TCTGGCCAGCTAGCTCCAT |
| Gapdh-ChipF | CTCTGCTCCTCCCGTTTCCA |
| Gapdh-ChipR | CCCACTCCGCGATTTTCA |
| RMCEupmf | TCTTGGAAGAGAAACTCTTAGGG |
| RMCEdomr | TGTATACAGATCTACCAACATTACGA |
| SnrpnGR2 | CAAAGGATTGCTCACCAATTCTC |
| SnrpnGF2 | GTGAAAGTGCATCCTATTTGACC |
| TOPL1pBF1 | TTYGGGTTGTAGGAATTYGATATTTGTAG |
| TOPL1pBR1 | CTAATAACRACCRCCAATATACTAAAATTC |
| SnrpnBF2 | GATTAAAATATTTTAGATTTGGGTTTATTAAG |

**Electrophoretic Mobility Shift Assay (EMSA) probes**

| Double-stranded oligonucleotide probes | Sequence |
| --- | --- |
| Control | 5’-GTAATATTGCCGCAGTAATA-3’  3’-CATTATAACGGCGTCATTAT-5’ |
| Methylated Control | 5’-GTAATATTGCZGCAGTAATA-3’  3’-CATTATAACGGZGTCATTAT-5’ |
| 1A | 5’-GTAATAT**A**GCCGCAGTAATA-3’  3’-CATTATA**T**CGGCGTCATTAT-5’ |
| Methylated 1A | 5’-GTAATAT**A**GCZGCAGTAATA-3’  3’-CATTATA**T**CGGZGTCATTAT-5’ |
| 1C | 5’-GTAATAT**C**GCCGCAGTAATA-3’  3’-CATTATA**G**CGGCGTCATTAT-5’ |
| Methylated 1C | 5’-GTAATAT**C**GCZGCAGTAATA-3’  3’-CATTATA**G**CGGZGTCATTAT-5’ |
| Fully Methylated 1C | 5’-GTAATAT**Z**GCZGCAGTAATA-3’  3’-CATTATA**G**ZGGZGTCATTAT-5’ |
| 1G | 5’-GTAATAT**G**GCCGCAGTAATA-3’  3’-CATTATA**C**CGGCGTCATTAT-5’ |
| Methylated 1G | 5’-GTAATAT**G**GCZGCAGTAATA-3’  3’-CATTATA**C**CGGZGTCATTAT-5’ |
| 2A | 5’-GTAATATT**A**CCGCAGTAATA-3’  3’-CATTATAA**T**GGCGTCATTAT-5’ |
| Methylated 2A | 5’-GTAATATT**A**CZGCAGTAATA-3’  3’-CATTATAA**T**GGZGTCATTAT-5’ |
| 2C | 5’-GTAATATT**C**CCGCAGTAATA-3’  3’-CATTATAA**G**GGCGTCATTAT-5’ |
| Methylated 2C | 5’-GTAATATT**C**CZGCAGTAATA-3’  3’-CATTATAA**G**GGZGTCATTAT-5’ |
| 2T | 5’-GTAATATT**T**CCGCAGTAATA-3’  3’-CATTATAA**A**GGCGTCATTAT-5’ |
| Methylated 2T | 5’-GTAATATT**T**CZGCAGTAATA-3’  3’-CATTATAA**A**GGZGTCATTAT-5’ |
| 3A | 5’-GTAATATTG**A**CGCAGTAATA-3’  3’-CATTATAAC**T**GCGTCATTAT-5’ |
| Methylated 3A | 5’-GTAATATTG**A**ZGCAGTAATA-3’  3’-CATTATAAC**T**GZGTCATTAT-5’ |
| 3G | 5’-GTAATATTG**G**CGCAGTAATA-3’  3’-CATTATAAC**C**GCGTCATTAT-5’ |
| Methylated 3G | 5’-GTAATATTG**G**ZGCAGTAATA-3’  3’-CATTATAAC**C**GZGTCATTAT-5’ |
| 3T | 5’-GTAATATTG**T**CGCAGTAATA-3’  3’-CATTATAAC**A**GCGTCATTAT-5’ |
| Methylated 3T | 5’-GTAATATTG**T**ZGCAGTAATA-3’  3’-CATTATAAC**A**GZGTCATTAT-5’ |
| 5A | 5’-GTAATATTGCC**A**CAGTAATA-3’  3’-CATTATAACGG**T**GTCATTAT-5’ |
| 5C | 5’-GTAATATTGCC**C**CAGTAATA-3’  3’-CATTATAACGG**G**GTCATTAT-5’ |
| 5T | 5’-GTAATATTGCC**T**CAGTAATA-3’  3’-CATTATAACGG**A**GTCATTAT-5’ |
| 6A | 5’-GTAATATTGCCG**A**AGTAATA-3’  3’-CATTATAACGGC**T**TCATTAT-5’ |
| Methylated 6A | 5’-GTAATATTGCZG**A**AGTAATA-3’  3’-CATTATAACGGZ**T**TCATTAT-5’ |
| 6G | 5’-GTAATATTGCCG**G**AGTAATA-3’  3’-CATTATAACGGC**C**TCATTAT-5’ |
| Methylated 6G | 5’-GTAATATTGCZG**G**AGTAATA-3’  3’-CATTATAACGGZ**C**TCATTAT-5’ |
| 6T | 5’-GTAATATTGCCG**T**AGTAATA-3’  3’-CATTATAACGGC**A**TCATTAT-5’ |
| Methylated 6T | 5’-GTAATATTGCZG**T**AGTAATA-3’  3’-CATTATAACGGZ**A**TCATTAT-5’ |
| Commd1 | 5’-GTAACTGCGGCCGCCCCACA-3’  3’-CATTGACGCCGGCGGGGTGT-5’ |
| Methylated Commd1 | 5’-GTAACTGZGGCZGCCCCACA-3’  3’-CATTGACGZCGGZGGGGTGT-5’ |
| Ston2 B6 | 5’-CTGGGGCGGCAGTTGCGGCAGATGG-3’  3’-GACCCCGCCGTCAACGCCGTCTACC-5’ |
| Methylated Ston2 B6 | 5’-CTGGGGZGGCAGTTGZGGCAGATGG-3’  3’-GACCCZGCCGTCAAZGCCGTCTACC-5’ |
| Ston2 JF1 | 5’-CTGGGGCGGCAGTTGCCGCAGATGG-3’  3’-GACCCCGCCGTCAACGGCGTCTACC-5’ |
| Methylated Ston2 JF1 | 5’-CTGGGGZGGCAGTTGCZGCAGATGG-3’  3’-GACCCCGZCGTCAACGGZGTCTACC-5’ |

The hexameric ZFP57 target sequence is underlined. Hexamer base mutations are in bold. The 5-mC is indicated with Z
